# Dietary Behaviours Mediate Intervention Effects on Body Composition Outcomes in Children: Evidence from a Cluster-Randomised Controlled Trial

**DOI:** 10.3390/nu18183051

**Published:** 2026-09-18

**Authors:** Li Zhang, Lu Wang, Chun-Jie Yin, Yu-Qi Duan, Jiaolong Ma, Shi-Yu Yan, Yit Siew Chin, Yan Li, Hai-Jun Wang

**Affiliations:** 1 Clinical Research Center for Children’s Health and Diseases, Key Laboratory of Birth Regulation and Control Technology of National Health Commission of China, Shandong Provincial Maternal and Child Health Care Hospital Affiliated to Qingdao University, Jinan 250014, China; zhangljg83@163.com (L.Z.); liyanxj@sina.com (Y.L.); 2 School of Public Health, Shihezi University, Shihezi 832000, China; majiaolong@shzu.edu.cn; 3 National Health Commission Key Laboratory of Reproductive Health, Department of Maternal and Child Health, School of Public Health, Peking University, Beijing 100191, China; wangluwest@outlook.com (L.W.); yinchunjie@bjmu.edu.cn (C.-J.Y.); 18811318259@163.com (Y.-Q.D.); yan_sy@bjmu.edu.cn (S.-Y.Y.); 4 Department of Nutrition, Faculty of Medicine and Health Sciences, Universiti Putra Malaysia, Serdang 43400, Malaysia; chinys@upm.edu.my

**Keywords:** children, obesity, body composition, dietary behaviour, principal component analysis, mediation analysis, randomised controlled trial

## Abstract

Background/Objectives: Few paediatric obesity trials have examined behavioural mediators of intervention effects on detailed body composition outcomes beyond body mass index (BMI). Given the complexity of multiple correlated body composition indicators, this study aimed to employ principal component analysis (PCA) to derive a composite adiposity factor and to apply mediation analysis to identify whether changes in dietary behaviours and physical activity mediated the effects of the DECIDE-Children intervention on body composition. Methods: This study included 1180 children from a 9-month cluster-randomised controlled trial that delivered a multifaceted, digitally assisted intervention targeting children (health education, physical activity reinforcement, and app-based BMI monitoring/feedback) and their environments (school food policies and family engagement). Outcomes were changes in body composition (whole-body and trunk fat mass, fat-free mass, and muscle mass indices measured by bioelectrical impedance analysis). Potential mediators included changes in dietary behaviour, screen time, and moderate-to-vigorous physical activity (MVPA), assessed via validated questionnaires. PCA was used to reduce the dimensionality of body composition variables and to derive a composite adiposity factor (PCA_1), with higher values indicating greater adiposity and lower muscle mass. Mediation was tested using linear mixed-effects models adjusted for age, sex, and region. Results: The intervention significantly reduced adiposity indicators (e.g., whole-body and trunk fat mass percentage decreased by 1.003%, and PCA_1 decreased by 0.942 SD) and concurrently increased fat-free mass percentage and muscle mass percentage at both whole-body and trunk levels. Dietary behaviour significantly mediated the intervention effects on all body composition outcomes (e.g., mediating 16.1% of the effect on PCA_1), whereas physical activity (both screen time and MVPA) showed no mediation. Sensitivity analyses supported the primary findings. Conclusions: The DECIDE-Children intervention improved body composition by reducing adiposity and increasing fat-free and muscle mass percentage, effects primarily mediated by dietary behaviour. These findings highlight dietary behaviour as a key component in paediatric obesity intervention.

## 1. Introduction

Childhood obesity has become a major global public health crisis. According to the latest United Nations Children’s Fund report, obesity now surpasses underweight among school-age children and adolescents worldwide for the first time [1]. Currently, one in five children and adolescents (approximately 391 million) are overweight, with nearly half meeting obesity criteria [1,2]. A spatial-temporal trend analysis covering 191 countries from 1975 to 2016 projects that by 2030, the global prevalence of overweight and obesity among those aged 5 to 19 years will reach 30.0% [3]. In China, this combined prevalence reached 37.0% in 2020 [4]. Excess adiposity in childhood strongly tracks into adulthood, increasing the risk of type 2 diabetes, hypertension, and psychosocial morbidity [5,6,7], and imposes growing economic costs for individuals and society [8]. The rising prevalence of childhood obesity is driven by a complex interplay of genetic, environmental, and behavioural factors, among which poor dietary habits and sedentary lifestyles that contribute to a positive energy balance play a critical role [1,7].

However, few childhood obesity trials have simultaneously examined how concurrent changes in multiple dietary behaviours and physical activity mediate intervention effects on both adiposity and muscle indicators beyond body mass index (BMI) [9]. Most existing mediation studies have focused on single behaviours (e.g., sugar-sweetened beverages or overall moderate-to-vigorous physical activity [MVPA]) and have used BMI or waist circumference as the primary adiposity outcome [10]. Importantly, BMI and waist circumference cannot distinguish fat mass from lean mass (or fat-free mass), nor do they capture regional fat distribution, yet these components are independent predictors of cardiometabolic risk in children [11,12]. Therefore, detailed body composition indices beyond whole-body measures, particularly trunk-specific fat and muscle mass, as well as composite adiposity indicators integrating multiple fat and muscle indices through specific statistical methods, may provide more holistic and clinically meaningful measures of obesity-risk reduction than whole-body measures alone. Moreover, the preservation or enhancement of muscle mass (or fat-free mass) during obesity interventions is increasingly recognised as a key indicator of metabolic health, yet it remains largely overlooked in mediation analyses within cluster-randomised controlled trials. Consequently, evaluating the mediating pathways that link lifestyle interventions to these refined body composition outcomes is essential to fully understand their mechanisms of action.

Bioelectrical impedance analysis (BIA) provides a practical and non-invasive alternative to reference methods such as dual-energy X-ray absorptiometry (DXA) for assessing body composition in paediatric populations. A recent systematic review of 28 studies concluded that multi-frequency segmental BIA devices show moderate to strong correlations with DXA for fat mass, with correlation coefficients ranging from 0.61 to 0.99 [13]. In a Chinese paediatric population, BIA showed strong correlations for fat mass (r = 0.95), fat-free mass (r = 0.98), and fat percentage (r = 0.86) [14]. These findings support the use of BIA in school-based obesity prevention trials. Furthermore, when multiple body composition parameters are assessed simultaneously, principal component analysis (PCA) offers a valuable approach to reduce these correlated measures into meaningful dimensions. For instance, a recent paediatric study applied PCA to eight BIA-derived parameters and successfully extracted two distinct components: a fat-free mass axis and an adiposity axis, demonstrating the utility of this method in capturing the underlying structure of body composition data in children with overweight and obesity [15].

The Diet, Exercise and Cardiovascular Health-Children (DECIDE-Children) study was a cluster-randomised controlled trial conducted in 24 primary schools across three Chinese cities [16]. The multicomponent intervention targeted children (health education lessons, physical activity reinforcement, and BMI feedback via a smartphone app) and their environments (school food policies and family engagement). Over the one-school-year trial, the intervention reduced BMI by 0.46 (95% CI: −0.67 to −0.25; *p* < 0.001) and reduced obesity prevalence by 27.0% relative to baseline, compared with 5.6% in the control group [17]. A previous mediation analysis using DECIDE-Children data showed that a composite score of dietary behaviour change mediated 13% to 17% of the intervention effect on BMI and body fat mass percentage [18]. Nevertheless, that study did not examine mediation for more granular body composition outcomes such as trunk fat mass (FM), fat-free mass (FFM) or muscle mass (MM) indices.

The present study aimed to quantify the mediating effects of concurrent changes in dietary and physical activity behaviours on body composition outcomes of the DECIDE-Children intervention, including whole-body, trunk, and PCA-derived composite indices assessed via BIA. We hypothesised that dietary and physical activity behaviours would mediate the intervention effects on adiposity, fat-free mass, and muscle mass indicators.

## 2. Materials and Methods

### 2.1. Study Design and Participants

DECIDE-Children was a cluster-randomised controlled trial conducted among 1392 grade 4 children (aged 8 to 10 years) from 24 schools across three Chinese cities: Beijing (northern China), Changzhi (central China), and Urumqi (western China). Schools were randomized 1:1 to intervention or control after stratification by district and region [16]. Overweight and obesity were defined using age- and sex-specific BMI percentiles based on Chinese reference values, as described in the main trial publication [17]. The present analysis included 1180 children with complete baseline and follow-up data on dietary behaviours, physical activity, and body composition. Participants were excluded for incomplete questionnaires or a BMI Z-score outside ± 3 SD: 86 from the intervention group (85 incomplete, 1 BMI outlier) and 96 from the control group (95 incomplete, 1 BMI outlier). The participant flow diagram has been published previously [18].

### 2.2. Intervention

The 9-month multifaceted intervention was based on the social-ecological model and comprised five components: child level: (1) classroom health education (10 sessions); (2) ≥1 h/day MVPA reinforcement during school; (3) monthly BMI monitoring and feedback via smartphone app; and environment level: (4) school policies restricting unhealthy foods and beverages; and (5) parent workshops and app-assisted family monitoring and goal-setting. Further details of the intervention have been published previously [16], and the primary outcomes are reported elsewhere [17].

### 2.3. Assessment of Body Composition, Dietary Behaviours, and Physical Activity

At both baseline and endpoint, trained staff measured height using calibrated stadiometers, and body composition was assessed using an eight-electrode, multi-frequency BIA (Tanita MC-780 MA, Tanita Corporation, Tokyo, Japan). BIA has been validated against DXA in Chinese children and adolescents aged 6 to 17 years, showing strong correlations for fat mass (r = 0.95), fat-free mass (r = 0.98), and fat percentage (r = 0.86) [14], supporting its use in this population, although the specific Tanita MC-780 MA device has not been individually validated. Impedance was recorded at 5, 50, and 250 kHz and translated into estimates of fat mass (FM, kg), fat-free mass (FFM, kg), muscle mass (MM, kg), and their regional distributions using device-specific equations validated against DXA [19,20]. Whole-body outputs included BMI, FM, FM%, FFM, and MM; segmental results provided corresponding values for the trunk, arms, and legs. Derived indices included FFM% = (FFM/body weight) × 100, MM% = (MM/body weight) × 100, fat mass index (FMI) = FM/height^2^ (kg/m^2^), fat-free mass index (FFMI) = FFM/height^2^ (kg/m^2^), consistent with current literature [21,22,23]. For segmental analysis, we focused on trunk FM (kg), trunk FM%, trunk FFM (kg), trunk FFM%, trunk MM (kg), and trunk MM%.

Dietary behaviours and physical activity were assessed using validated questionnaires. Five dietary behaviours, including consumption of sugar-sweetened beverages, fried food, Western fast food, unhealthy snacks, and eating out, were dichotomised as meeting versus not meeting recommendations, and a composite dietary behaviour change score (0–5) was calculated. Screen time was dichotomised as <1 h/day versus ≥1 h/day, and MVPA as ≥60 min/day versus <60 min/day, using established cut-offs [24,25]. Further details of the questionnaires have been published previously [16,17].

### 2.4. Statistical Analyses

The continuous variables were presented as means ± SD and the categorical variables as *n* (%). Between-group differences were examined with Student’s *t*-tests (continuous variables) and Chi-square tests (categorical variables). Body composition variables were Z-standardised and subjected to principal component analysis (covariance matrix). Components explaining ≥ 90% of cumulative variance were retained.

Body composition metrics were categorised into two domains: whole body and trunk. Whole-body metrics included (1) BMI (kg/m^2^); (2) body fat mass indices: ① body FM (kg), ② body FM% (%), and ③ body FMI (kg/m^2^); (3) body fat-free mass indices: ① body FFM (kg), ② body FFM% (%), and ③ body FFMI (kg/m^2^); and (4) body muscle mass indices: ① body MM (kg) and ② body MM% (%). Trunk metrics included: (1) Trunk fat mass indices: ① trunk FM (kg) and ② trunk FM% (%); (2) trunk fat-free mass indices: ① trunk FFM (kg) and ② trunk FFM% (%); and (3) trunk muscle mass indices: ① trunk MM (kg) and ② trunk MM% (%). From these, four whole-body variables (BMI, body FM%, body FFM%, and body MM%) and three trunk variables (trunk FM%, trunk FFM%, and trunk MM%) were selected for principal component analysis (PCA) to reduce dimensionality.

Intervention effects on whole-body and trunk body composition indices, PCA-derived scores, and the three candidate mediators (dietary behaviour, screen time, and MVPA) were estimated using linear mixed effects models with a random intercept at the school level to account for clustering of children within schools. Univariate models included only the intervention indicator; multivariate models were additionally adjusted for age, sex, and region.

Mediation was assessed using the product-of-coefficients approach. Path A: intervention → mediator change; Path B: mediator change → outcome change (adjusted for intervention); Path C: intervention → outcome change (total and direct). Indirect effects (A × B) and the proportion mediated (indirect/[indirect + direct]) were estimated with 1000 bootstrap resamples, adjusting for age, sex, and region.

The mediation effect analysis showed that MVPA did not mediate the intervention effects. However, given that MVPA might still influence changes in body composition, we additionally adjusted all multivariate models for MVPA and re-estimated the intervention effects on body composition outcomes and the two retained mediators (dietary and screen behaviours), as well as their mediation effects.

All analyses were performed in R 4.3.2 software. Statistical significance was set at *p* < 0.05 (two-tailed).

## 3. Results

### 3.1. Participant Characteristics

Participant characteristics at baseline are summarised in Table 1. Among the 1180 enrolled children, 600 (50.8%) were in the intervention group and 580 (49.2%) in the control group. Mean age (9.63 ± 0.36 years), sex distribution (girls 48.6%), and regional composition (Beijing 35.0%, Changzhi 27.1%, and Urumuqi 37.9%) did not differ between two groups (all *p* > 0.05).

Whole-body and trunk body composition indices were likewise comparable between groups at baseline. BMI, adiposity indicators (FM, FM%, FMI, trunk FM, and trunk FM%), and lean/muscle indicators (FFM, FFM%, FFMI, MM, and MM%) showed no statistically significant differences between the two groups (all *p* > 0.05).

### 3.2. Dimension Reduction of Body Composition Outcomes

**Figure 1:**
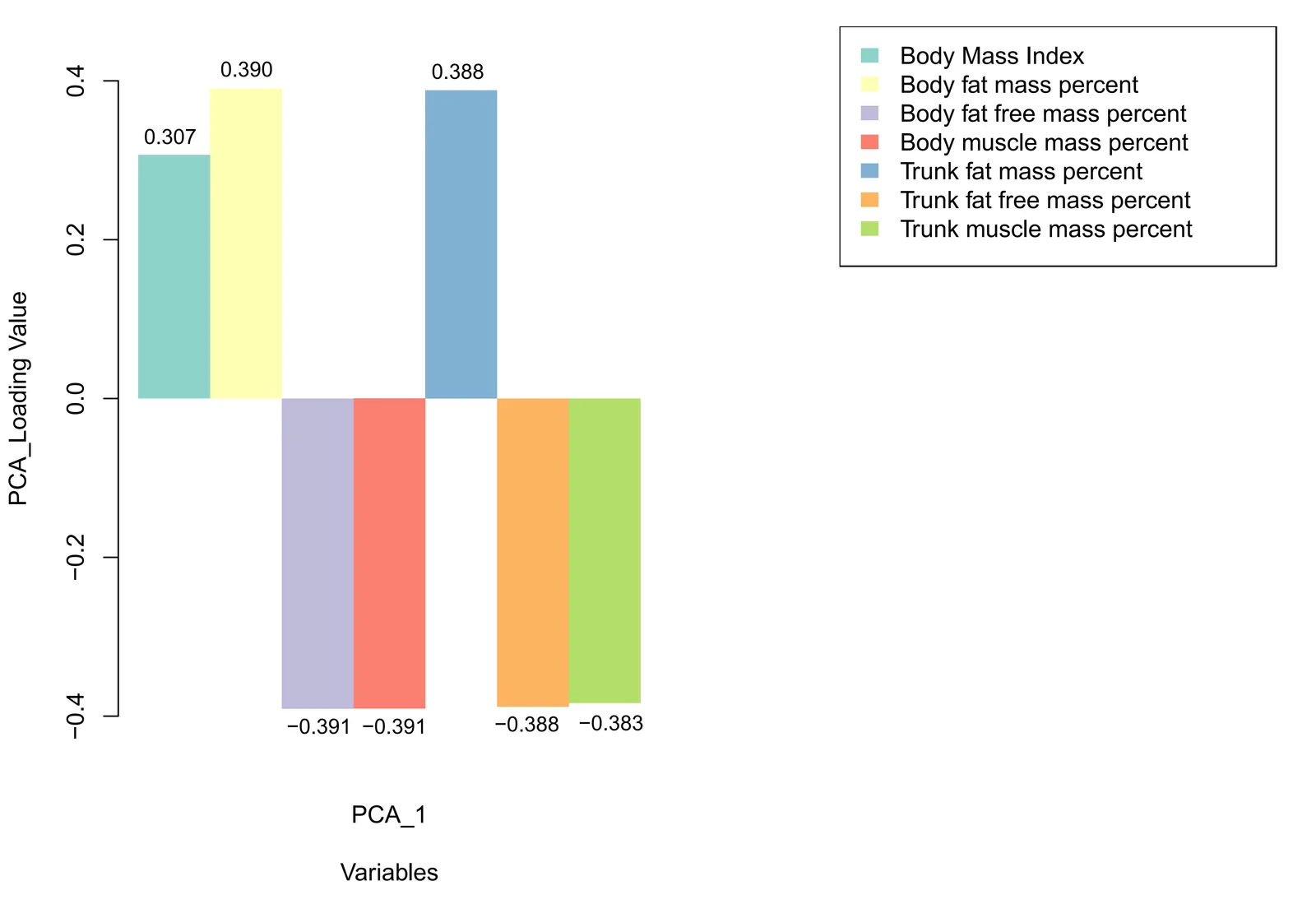
PCA loading plot of body composition indicators. Notes: Principal component analysis (PCA) was performed to reduce the dimensionality of body composition variables. PCA_1 represents the primary axis of variance in body composition. The loading coefficients for PCA_1 are calculated as follows: PCA_1 = 0.307 × Body Mass Index + 0.390 × Body fat mass percentage − 0.391 × Body fat-free mass percentage − 0.391 × Body muscle mass percentage + 0.388 × Trunk fat mass percentage − 0.388 × Trunk fat-free mass percentage − 0.383 × Trunk muscle mass percentage.

The first principal component (PCA_1) explained 91.32% of the variance (Figure 1). PCA_1 loaded positively on adiposity indices (BMI, body FM%, and trunk FM%) and negatively on fat-free and muscle mass indices (body and trunk FFM% and MM%). Lower PCA_1 scores therefore indicate lower adiposity and higher lean mass and muscle mass.

### 3.3. Intervention Effects on Body Composition Indicators and Candidate Mediators

Linear mixed effects models revealed consistent favourable effects of the DECIDE-Children intervention (Table 2). After adjustment for age, sex, and region, the children in the intervention group exhibited significant reductions in whole-body adiposity: BMI decreased by 0.399 kg/m^2^ (95% CI: −0.609 to −0.189; *p* = 0.002), FM by 0.650 kg (95% CI: −0.996 to −0.304; *p* = 0.002), FM% by 1.003% (95% CI: −1.625 to −0.380; *p* = 0.006), and FMI by 0.295 kg/m^2^ (95% CI: −0.445 to −0.144; *p* = 0.001). Parallel improvements were observed at the trunk level, with trunk FM decreasing by 0.301 kg (95% CI: −0.486 to −0.116; *p* = 0.005) and trunk FM% by 1.003% (95% CI: −1.733 to −0.273; *p* = 0.016).

Conversely, relative lean and muscle parameters increased: whole-body FFM% and MM% rose by 0.999% and 0.010%, respectively (*p* < 0.01), whereas absolute FFM and MM remained statistically unchanged. The composite adiposity factor (PCA_1) declined by 0.942 SD (95% CI: −1.508 to −0.376; *p* = 0.005).

Among the candidate mediators, the intervention significantly improved dietary behaviour (β = 1.074; 95% CI: 0.763 to 1.385; *p* < 0.001) and reduced the proportion of children exceeding 1 h/day of recreational screen time (OR = 1.867; 95% CI: 1.309 to 2.662; *p* = 0.001), whereas MVPA remained unchanged (OR = 1.019; *p* = 0.916). Using continuous MVPA (hours/week) as the outcome, the between-group difference was also non-significant (β = 0.345, 95% CI: −1.093 to 1.793, *p* = 0.643), consistent with the binary analysis (Table 2).

### 3.4. Mediation Analyses

#### 3.4.1. Mediation Effects of Dietary Behaviours

**Figure 2:**
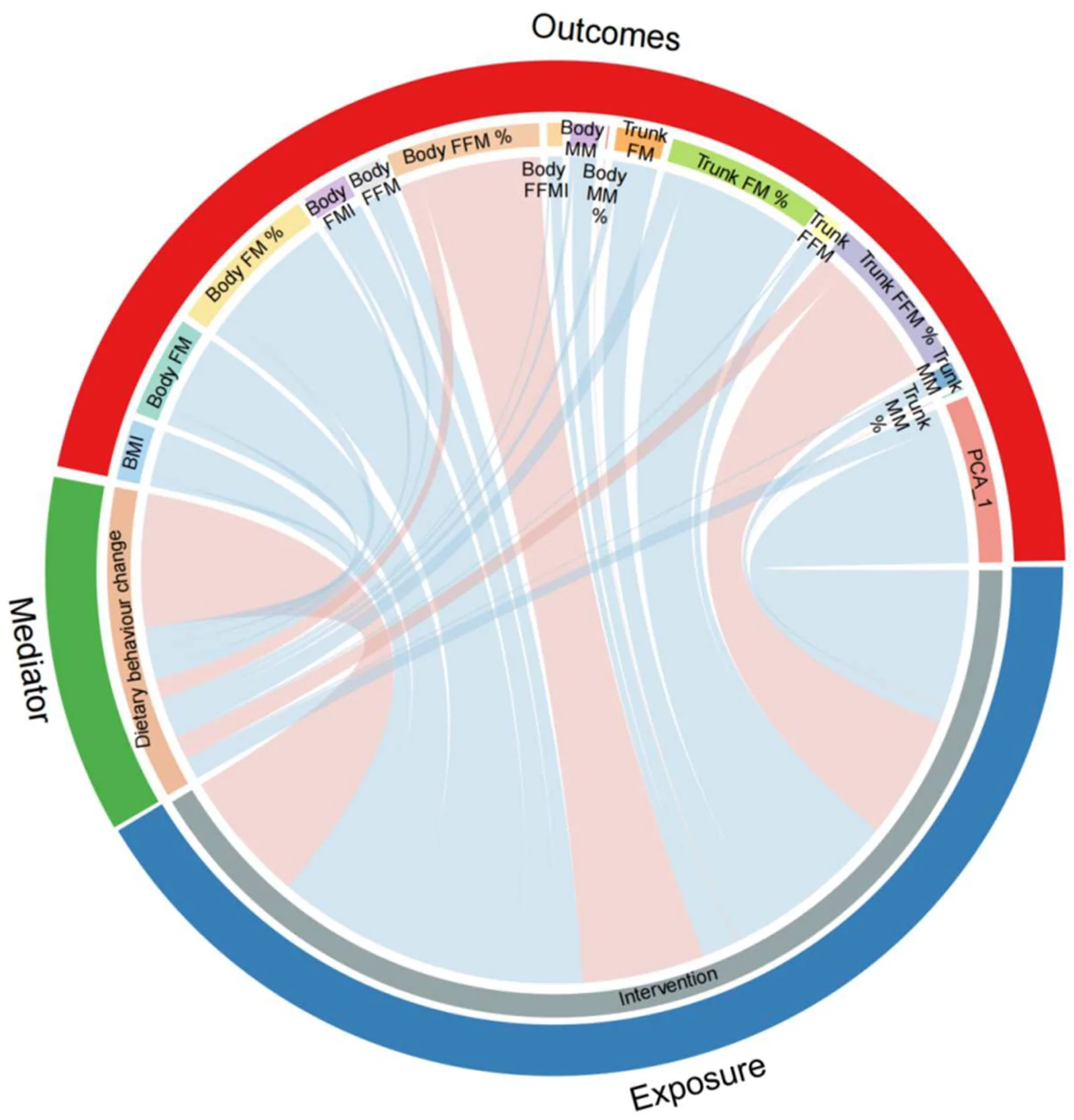
Chord diagram of mediation analysis. Note: The outermost coloured arcs represent the exposure (blue), mediators (green), and outcomes (red), respectively. The inner sectors correspond to specific variables. The colours of the connecting lines indicate the direction of the effects between variables, with red representing positive associations and blue representing negative associations. The width of the connecting lines reflects the magnitude of the effect sizes.

Improvements in composite dietary behaviours statistically mediated the intervention’s beneficial effect on body composition outcomes (Table 3, Figure 2). After adjusting for age, sex, and region, the indirect (mediated) effects were significant for the reduction of all whole-body adiposity indicators: BMI (β = −0.054 kg/m^2^; 95% CI: −0.098 to −0.016; *p* = 0.006), body FM (β = −0.109 kg; 95% CI: −0.192 to −0.040; *p* = 0.002), body FM% (β = −0.154%; 95% CI: −0.290 to −0.039; *p* = 0.008), and body FMI (β = −0.047 kg/m^2^; 95% CI: −0.086 to −0.015; *p* = 0.004). Significant mediated effects were also observed for trunk fat reduction: trunk FM (β = −0.063 kg; 95% CI: −0.110 to −0.024; *p* <0.001) and trunk FM% (β = −0.183%; 95% CI: −0.341 to −0.048; *p* = 0.006). Dietary behaviours also mediated the reduction in the composite adiposity factor (PCA_1) by 0.152 SD (95% CI: −0.282 to −0.044; *p* = 0.006). Concurrently, dietary behaviour mediated increases in lean and muscle parameters: body FFM% (β = 0.153%; 95% CI: 0.036 to 0.282), body MM% (β = 0.001%; 95% CI: 0.000 to 0.003), and their trunk counterparts (all *p* < 0.05).

The proportion of the total intervention effect mediated by dietary change ranged from 13% (for BMI) to 21% (for trunk FM), with a mean of 17% across adiposity outcomes. Overall, concurrent improvements in dietary behaviour consistently mediated the intervention’s favourable body composition changes.

#### 3.4.2. Mediating Effects of Screen Time Behaviour

Screen time behaviour demonstrated no significant mediating effects for any body composition measures, including PCA_1 (Table 4).

### 3.5. Sensitivity Analyses

To assess the robustness of the null MVPA mediation findings, we examined the intervention effect on accelerometer-measured in-school MVPA (minutes/hour, weekdays 8:00 a.m. to 4:59 p.m.) as a continuous variable in the subsample (*n* = 88) that met the inclusion criteria (Supplementary Materials File S1: Analysis of the accelerometer subsample). Adjusted for age, sex, and region, the between-group difference was not significant (β = 0.4267, 95% CI: −0.411 to 1.264, *p* = 0.247), consistent with the findings based on self-reported MVPA. Furthermore, the results of sensitivity analyses adjusting for MVPA remained consistent with the primary analyses (Supplementary Materials Tables S1 and S2).

## 4. Discussion

### 4.1. Overview of Intervention Effects on Body Composition

This study employed multi-frequency BIA to assess a comprehensive set of body composition indicators, including whole-body and segmental fat mass, fat-free mass, and muscle mass indices. The findings extend beyond a simple reduction in BMI, revealing that the intervention produced favourable changes across multiple body composition domains. Specifically, the intervention led to significant decreases in adiposity indicators alongside concurrent increases in muscle mass and lean mass percentage. It is important to note that while the relative percentages of fat-free and muscle mass increased, their absolute values (kg) did not change significantly. This suggests that the observed increase in percentages is primarily attributable to the reduction in total body mass and fat mass in the intervention group, rather than to a true gain in lean or muscle tissue. Therefore, the improvement in body composition should be interpreted as a favourable shift in the proportion of fat-free mass relative to total mass, rather than as an increase in muscle mass per se. This overall beneficial shift was further supported by the PCA-derived composite body composition indicator (PCA_1), which showed a significant reduction (β = −0.942, 95% CI: −1.508 to −0.376, *p* = 0.005), indicating a global improvement in body composition health. Moreover, beyond overall improvements in whole-body composition, the intervention also produced favourable changes in trunk body composition, including significant reductions in trunk fat mass percentage and concurrent increases in trunk fat-free mass percentage and muscle mass percentage. Given the independent predictive value of central obesity for metabolic outcomes, these results underscore the importance of utilising multidimensional body composition assessment to fully capture the health benefits of paediatric obesity interventions.

### 4.2. Whole-Body Composition Improvements in the Intervention

Our findings align with several recent cluster-randomised controlled trials that have examined the effects of lifestyle interventions on detailed body composition outcomes. A network meta-analysis of 91 RCTs involving 58,649 children confirmed that multicomponent lifestyle interventions significantly reduced both BMI Z-score (MD = −0.11, 95% CI: −0.18 to −0.04) and body fat percentage (MD = −1.69%, 95% CI: −2.97 to −0.42) compared to usual care, reinforcing the value of comprehensive intervention approaches [26]. However, that meta-analysis did not analyse muscle or lean mass indicators, leaving a gap in understanding how interventions affect these important components of body composition.

Notably, not all studies have observed consistent effects. In contrast to our findings, a cluster-randomised trial among Malaysian adolescents aged 13 to 16 years reported that the MyBFF@school multicomponent intervention did not lead to significant changes in BMI Z-score, body fat percentage, or skeletal muscle mass after six months [27]. Similarly, the KaziAfya trial among Tanzanian schoolchildren found no statistically significant differences in overall body composition between intervention and control groups. However, it reported sex-specific effects of physical activity and micronutrient supplementation on FM and FFM, further emphasizing the need for detailed body composition assessment to understand differential intervention responses [28].

Our study findings support the notion that interventions targeting multiple lifestyle components can induce favourable changes in body composition, particularly reducing adiposity while preserving or increasing MM%, which are not adequately captured by traditional anthropometric indices.

### 4.3. Trunk Body Composition Improvements in the Intervention

The importance of assessing regional body composition, especially trunk fat distribution, has been increasingly recognised in the literature. Central obesity, characterised by excess trunk fat accumulation, is an independent and powerful predictor of cardiometabolic risk in both adults and children [29,30]. Accumulating evidence suggests that trunk fat mass, rather than total body fat, is more strongly associated with insulin resistance, dyslipidaemia, and inflammatory markers in paediatric populations [31,32]. Therefore, interventions that specifically target trunk adiposity may confer greater metabolic health benefits than those merely reducing overall body fat.

Several recent studies have reported on regional body composition changes following lifestyle interventions in children and adolescents. A multidisciplinary weight loss programme among adolescents with severe obesity demonstrated that the steepest drop in fat mass occurred in the trunk region, with reductions of 10.1% in boys and 11.4% in girls over 6 to 12 months [33], suggesting that trunk fat may be more responsive to lifestyle modification compared to peripheral fat depots. Additionally, a family-centred lifestyle intervention among 9 to 12 year old children with overweight and obesity found that while the intervention resulted in only modest reductions in BMI Z-scores, it successfully prevented the increase in trunk fat mass observed in the control group over 12 months [34]. A recent study comparing lifestyle modification with and without pharmacotherapy reported that lifestyle modification alone reduced trunk fat percentage by 1.90% over 12 months [35], further supporting that trunk fat is modifiable through behavioural approaches.

Given the strong predictive value of central obesity for cardiovascular disease in later life [29,30], the observed improvements in trunk body composition carry substantial public health significance and add to the growing evidence that school-based interventions can effectively mitigate central adiposity in childhood. These findings also have practical implications for school and community nursing: the reduction in trunk fat, mediated primarily by dietary behaviour change, supports prioritising nutrition education within obesity prevention programmes.

### 4.4. Mediating Roles of Dietary and Physical Activity Behaviours

Dietary behaviour represents one of the most proximal and modifiable determinants of energy balance and body composition in children. Unlike physical activity, which often shows modest and inconsistent changes in school-based interventions, dietary behaviours may be more responsive to targeted intervention strategies and may exert more direct effects on both fat reduction and muscle preservation [18]. The mediation pattern observed in this study reflects a critical physiological mechanism: by reducing adiposity, the intervention concurrently increased the proportion of muscle mass and fat-free mass, with dietary behaviour change serving as a key pathway. The proportion of the intervention effect on adiposity indicators mediated by dietary behaviour ranged from 13.3% to 21.0%. Our observation that dietary behaviour change contributed to trunk fat reduction aligns with evidence from a prospective study among Thai children, which reported that higher total protein intake was correlated with a decrease in trunk fat mass index, and dietary fibre intake was negatively correlated with visceral fat area [36].

Several biological mechanisms may explain these findings. First, reducing intake of energy-dense foods directly lowers total energy intake, creating the negative energy balance necessary for fat loss. Second, increasing protein and fibre intake supports muscle protein synthesis and promotes satiety [36]. Third, dietary changes may favourably influence metabolic hormones and inflammatory markers, improving the metabolic environment for both fat oxidation and muscle protein accretion [37]. Notably, dietary behaviour mediated up to one-fifth of the intervention effect on trunk fat, underscoring the potential of nutrition-focused strategies to mitigate central adiposity in childhood.

In contrast to dietary behaviour, neither screen time nor MVPA emerged as significant mediators. The intervention improved adherence to screen time recommendations but did not significantly increase MVPA as measured by questionnaire. To address whether dichotomising MVPA (meeting vs. not meeting guidelines) may have masked changes in physical activity levels, we repeated the mediation analysis using continuous MVPA (hours/week) as the mediator. The results showed no significant between-group difference for continuous MVPA (β = 0.345, 95% CI: −1.093 to 1.793, *p* = 0.643), consistent with the binary analysis (OR = 1.019, 95% CI: 0.718 to 1.446, *p* = 0.916). These findings confirm that the intervention did not significantly alter MVPA levels regardless of whether MVPA was treated as a binary or continuous variable.

Previous accelerometer-based findings from this trial revealed a more nuanced pattern: the intervention modestly increased MVPA during in-school periods but did not improve out-of-school physical activity [38]. To further explore this, we conducted an additional analysis restricted to the accelerometer subsample that met the inclusion criteria for the present mediation analysis. Using the same covariates (age, sex, and region), we found no significant intervention effect on in-school MVPA in this subsample (β = 0.427, 95% CI: −0.411 to 1.264, *p* = 0.247; see Supplementary Materials), indicating that the previously detected increase did not reach statistical significance in this reduced sample. These findings are consistent with the null mediation results based on self-reported MVPA and suggest that even though in-school MVPA slightly increased, the overall MVPA dose, particularly during out-of-school hours, may have been insufficient to induce detectable improvements in body composition.

Regarding screen time, despite improvements in adherence, no significant mediating effects on body composition were observed. A plausible explanation is that reductions in screen time do not necessarily translate into increased MVPA; rather, they may be accompanied by increases in light-intensity physical activity (LPA) [39]. However, longitudinal evidence from school-aged children suggests that LPA is not significantly associated with changes in adiposity or cardiorespiratory fitness over time, whereas only MVPA, and particularly vigorous physical activity (VPA), demonstrates significant favourable associations with these outcomes [40]. Thus, reductions in screen time, in the absence of a concomitant increase in MVPA, would be expected to produce only marginal changes in body composition, effects likely too small to be detected within the current sample size.

Of note, despite the lack of significant mediation by movement behaviours, the intervention yielded significant improvements in whole-body and trunk body composition. Furthermore, previous findings from this trial have demonstrated significant gains in physical fitness, such as increased one-minute rope-jump counts [17], suggesting that the intervention may have improved physical function through pathways not captured by MVPA and screen time alone. The apparent discrepancy warrants further investigation. One potential explanation is that the increase in in-school MVPA detected in a previous study from this trial [38], while below the threshold required to mediate detectable changes in body composition, may have nonetheless contributed to improvements in physical fitness. This possibility aligns with the notion that fitness adaptations may be more sensitive to changes in MVPA than body composition measures, particularly over the intervention period examined.

### 4.5. Strengths and Limitations

A key methodological strength of this study is its cluster-randomised controlled trial design, which minimises selection bias and enhances internal validity. Multi-frequency BIA enabled detailed assessment of whole-body, segmental, and PCA-derived composite body composition indices, moving beyond BMI. Simultaneously examining the mediating roles of dietary behaviour, screen time, and physical activity further strengthened the study by elucidating behavioural mechanisms underlying health improvements.

The findings carry important implications for clinical practice and health policy. First, they provide evidence that the intervention confers health benefits beyond BMI reduction, particularly in improving overall body composition and mitigating central obesity, a key predictor of future cardiometabolic risk. Second, the beneficial effects were primarily mediated by changes in dietary behaviours rather than physical activity, underscoring the pivotal role of dietary modification in childhood obesity interventions. These results are particularly relevant given the challenges of managing childhood obesity in diverse populations; our study, conducted across three geographically diverse Chinese cities, demonstrates that a school-based multicomponent intervention can be effective in a large cohort with significant regional diversity [41]. This is noteworthy because lifestyle interventions in minority ethnic groups have often shown limited effects on BMI [42]. The success of our approach, which produced measurable improvements in trunk fat reduction, suggests that such strategies hold promise for improving adiposity and metabolic health in other high-risk populations when adapted to local cultural contexts. From a clinical guideline perspective, our results support the inclusion of detailed body composition assessments beyond BMI in future recommendations and underscore the need for school-based policies that prioritise dietary behaviour modification, while recognising that physical activity promotion remains important for other health outcomes such as physical fitness and cardiovascular health.

Several limitations should be acknowledged. First, changes in physical activity behaviours, including screen time and MVPA, did not significantly mediate improvements in body composition, which may be attributable to the intervention’s modest impact on these behaviours [43]. Although the intervention improved screen time adherence and in-school MVPA, the lack of concomitant improvement in out-of-school MVPA may have precluded the detection of significant mediation effects. Second, the assessment of dietary behaviour and physical activity relied on validated self-report questionnaires, which are susceptible to recall and social desirability bias. Such measurement error may have attenuated the estimated mediation effects, potentially underestimating the true indirect effects [44,45]. This is particularly relevant for physical activity, as self-reported MVPA is generally subject to greater measurement error, which may have contributed to the null mediating effect observed for this variable [45]. In contrast, our dietary assessment captured qualitatively distinct behavioural patterns rather than quantitative intake, which may reduce random measurement error [44]. Importantly, accelerometry-based MVPA measurement in a subsample of participants [38] yielded results consistent with those from the self-reported questionnaires, further supporting the credibility of our questionnaire-based findings. Nonetheless, future studies employing objective measures, such as accelerometry for physical activity and biomarker-based dietary assessment, would provide more precise estimates of the mediating pathways. Third, the 9-month follow-up period is insufficient to establish the long-term sustainability of the observed effects. Fourth, while BIA is practical for large-scale school-based trials and has demonstrated acceptable accuracy and test-retest reliability in paediatric populations [13,46], it has inherent limitations: BIA accuracy varies by device type and is influenced by hydration status and proprietary equations [13]. To mitigate this, we used change values in mediation models, which reduces between-individual variability.

## 5. Conclusions

This study demonstrates that a multifaceted, school-based obesity intervention improved body composition in primary school children, reducing adiposity while increasing fat-free and muscle mass percentage at both whole-body and trunk levels, with the PCA-derived composite indicator also showing significant improvement. Mediation analyses identified improvements in dietary behaviour, rather than changes in physical activity, as the primary pathway underlying these effects. These findings underscore the importance of integrating dietary behaviour components into multicomponent obesity prevention programmes. The generalisability of these findings should be considered in light of the use of bioelectrical impedance analysis, questionnaire-based behavioural measures, and the specific age group and school setting. Future research should explore whether similar mediation patterns are observed using objective measures and in other populations.

## Figures and Tables

**Table 1:** Baseline characteristics of participants.

Characteristic	All (*N* = 1180)	Control Group (*n* = 580)	Intervention Group (*n* = 600)
Age (year), mean ± SD	9.63 ± 0.36	9.64 ± 0.37	9.62 ± 0.35
Gender, *n* (%)			
Girls	573 (48.56%)	274 (47.24%)	299 (49.83%)
Boys	607 (51.44%)	306 (52.76%)	301 (50.17%)
Region, *n* (%)			
Beijing	413 (35.00%)	197 (33.97%)	216 (36.00%)
Changzhi, Shanxi Province	320 (27.12%)	155 (26.72%)	165 (27.50%)
Urumuqi, Xinjiang Province	447 (37.88%)	228 (39.31%)	219 (36.50%)
Whole-body composition, mean ± SD			
(1) BMI (kg/m^2^)	18.67 ± 3.68	18.77 ± 3.63	18.57 ± 3.73
(2) Body fat mass indices			
① Body FM (kg)	8.45 ± 6.37	8.52 ± 6.21	8.38 ± 6.52
② Body FM% (%)	20.86 ± 10.14	21.04 ± 10.03	20.69 ± 10.25
③ Body FMI (kg/m^2^)	4.19 ± 2.91	4.23 ± 2.85	4.15 ± 2.97
(3) Body fat-free mass indices			
① Body FFM (kg)	27.85 ± 3.89	27.90 ± 3.84	27.80 ± 3.94
② Body FFM% (%)	79.13 ± 10.14	78.96 ± 10.03	79.31 ± 10.26
③ Body FFMI (kg/m^2^)	14.16 ± 1.06	14.20 ± 1.04	14.12 ± 1.09
(4) Body muscle mass indices			
① Body MM (kg)	26.41 ± 3.63	26.46 ± 3.57	26.36 ± 3.68
② Body MM% (%)	0.75 ± 0.10	0.75 ± 0.10	0.75 ± 0.10
Trunk body composition, mean ± SD			
(1) Trunk fat mass indices			
① Trunk FM (kg)	3.87 ± 3.16	3.94 ± 3.19	3.80 ± 3.12
② Trunk FM% (%)	17.82 ± 10.82	18.05 ± 10.86	17.59 ± 10.79
(2) Trunk fat-free mass indices			
① Trunk FFM (kg)	15.44 ± 2.00	15.45 ± 1.93	15.43 ± 2.07
② Trunk FFM% (%)	82.18 ± 10.82	81.95 ± 10.86	82.40 ± 10.79
(3) Trunk muscle mass indices			
① Trunk MM (kg)	14.64 ± 1.88	14.65 ± 1.80	14.64 ± 1.95
② Trunk MM% (%)	0.78 ± 0.10	0.78 ± 0.10	0.78 ± 0.10

Abbreviations: BMI, body mass index; FM, fat mass; FM%, fat mass percentage; FMI, fat mass index; FFM, fat-free mass; FFM%, fat-free mass percentage; FFMI, fat-free mass index; MM, muscle mass; MM%, muscle mass percentage; SD, standard deviation. Values are presented as mean ± SD or *n* (%). No statistically significant differences were observed in any baseline indicators between the intervention and the control groups (all *p* > 0.05; χ^2^ tests for categorical variables and Wilcoxon rank-sum tests for continuous variables).

**Table 2:** Effect of the intervention on body composition and candidate mediators.

Outcome	Univariate Model ^1^ β or OR (95% CI)	*p*	Multivariate Model ^2^ β or OR (95% CI)	*p*
Whole-body composition				
(1) BMI (kg/m^2^)	−0.397 (−0.612, −0.182)	0.001 **	−0.399 (−0.609, −0.189)	0.002 **
(2) Body fat mass indices				
① Body FM (kg)	−0.646 (−1.029, −0.263)	0.003 **	−0.650 (−0.996, −0.304)	0.002 **
② Body FM% (%)	−0.996 (−1.707, −0.284)	0.012 *	−1.003 (−1.625, −0.380)	0.006 **
③ Body FMI (kg/m^2^)	−0.291 (−0.467, −0.116)	0.004 **	−0.295 (−0.445, −0.144)	0.001 **
(3) Body fat-free mass indices				
① Body FFM (kg)	−0.192 (−0.398, 0.014)	0.082	−0.197 (−0.406, 0.011)	0.086
② Body FFM% (%)	0.992 (0.284, 1.701)	0.012 *	0.999 (0.379, 1.619)	0.006 **
③ Body FFMI (kg/m^2^)	−0.099 (−0.208, 0.010)	0.090	−0.099 (−0.204, 0.005)	0.084
(4) Body muscle mass indices				
① Body MM (kg)	−0.180 (−0.372, 0.012)	0.081	−0.185 (−0.380, 0.011)	0.086
② Body MM% (%)	0.009 (0.003, 0.016)	0.012 *	0.010 (0.004, 0.015)	0.005 **
Trunk body composition				
(1) Trunk fat mass indices				
① Trunk FM (kg)	−0.300 (−0.511, −0.089)	0.011 *	−0.301 (−0.486, −0.116)	0.005 **
② Trunk FM% (%)	−1.000 (−1.830, −0.169)	0.028 *	−1.003 (−1.733, −0.273)	0.016 *
(2) Trunk fat-free mass indices				
① Trunk FFM (kg)	−0.133 (−0.254, −0.012)	0.043 *	−0.137 (−0.256, −0.019)	0.038 *
② Trunk FFM% (%)	0.993 (0.163, 1.823)	0.029 *	0.996 (0.267, 1.726)	0.017 *
(3) Trunk muscle mass indices				
① Trunk MM (kg)	−0.132 (−0.248, −0.015)	0.038 *	−0.136 (−0.249, −0.023)	0.033 *
② Trunk MM% (%)	0.009 (0.001, 0.017)	0.036 *	0.009 (0.002, 0.016)	0.022 *
PCA-derived factors				
PCA_1	−0.938 (−1.581, −0.293)	0.009 **	−0.942 (−1.508, −0.376)	0.005 **
Candidate mediators				
Dietary behaviour change (0−5)	1.075 (0.766, 1.384)	<0.001 ***	1.074 (0.763, 1.385)	<0.001 ***
Screen time target attainment ^3^	1.870 (1.284, 2.771)	0.001 **	1.867 (1.309, 2.662)	0.001 **
MVPA target attainment ^3,4^	1.024 (0.667, 1.582)	0.912	1.019 (0.718, 1.446)	0.916

Abbreviations: BMI, body mass index; FM, fat mass; FM%, fat mass percentage; FMI, fat mass index; FFM, fat-free mass; FFM%, fat-free mass percentage; FFMI, fat-free mass index; MM, muscle mass; MM%, muscle mass percentage; MVPA, moderate-to-vigorous physical activity; PCA, principal component analysis. ^1^ Intervention indicator only. ^2^ Adjusted for age, sex, and region. ^3^ Dichotomised as 1 = meets guideline, 0 = does not meet guideline. ^4^ When MVPA was treated as a continuous variable (hours/week), the between-group difference was β = 0.345 (95% CI: −1.093 to 1.793, *p* = 0.643), consistent with the binary analysis. *** *p* < 0.001; ** *p* < 0.01; * *p* < 0.05.

**Table 3:** Mediating role of dietary behaviour change in the effects of the intervention on body composition.

Outcome	Indirect Effect ^1^ β (95% CI)	*p*	Direct Effect ^1^ β (95% CI)	*p*	Total Effect ^1^ β (95% CI)	*p*	% Mediated ^2^ (95% CI)
Whole-body composition							
(1) BMI (kg/m^2^)	−0.054 (−0.098, −0.016)	0.006 **	−0.345 (−0.560, −0.147)	<0.001 ***	−0.399 (−0.607, −0.196)	<0.001 ***	13.3 (4.0, 32.1)
(2) Body fat mass indices							
① Body FM (kg)	−0.109 (−0.192, −0.040)	0.002 **	−0.541 (−0.891, −0.215)	<0.001 ***	−0.650 (−0.985, −0.322)	<0.001 ***	16.7 (6.0, 38.4)
② Body FM% (%)	−0.154 (−0.290, −0.039)	0.008 **	−0.849 (−1.487, −0.257)	0.004 **	−1.003 (−1.621, −0.402)	<0.001 ***	15.3 (3.7, 42.6)
③ Body FMI (kg/m^2^)	−0.047 (−0.086, −0.015)	0.004 **	−0.247 (−0.400, −0.104)	<0.001 ***	−0.295 (−0.441, −0.152)	<0.001 ***	15.9 (5.0, 36.8)
(3) Body fat-free mass indices							
① Body FFM (kg)	−0.034 (−0.091, 0.018)	0.174	−0.163 (−0.388, 0.051)	0.150	−0.197 (−0.416, 0.013)	0.076	15.6 (−61.8, 143.5)
② Body FFM% (%)	0.153 (0.036, 0.282)	0.016 *	0.849 (0.212, 1.439)	0.002 **	1.001 (0.372, 1.603)	<0.001 ***	15.3 (3.4, 45.3)
③ Body FFMI (kg/m^2^)	−0.018 (−0.039, 0.001)	0.064	−0.082 (−0.194, 0.022)	0.144	−0.099 (−0.207, 0.006)	0.074	16.5 (−56.4, 144.8)
(4) Body muscle mass indices							
① Body MM (kg)	−0.031 (−0.084, 0.017)	0.178	−0.153 (−0.364, 0.047)	0.148	−0.184 (−0.389, 0.013)	0.076	15.2 (−59.6, 141.9)
② Body MM% (%)	0.001 (0.000, 0.003)	0.014 *	0.008 (0.002, 0.014)	0.002 **	0.010 (0.004, 0.015)	<0.001 ***	15.6 (3.6, 45.0)
Trunk body composition							
(1) Trunk fat mass indices							
① Trunk FM (kg)	−0.063 (−0.110, −0.024)	<0.001 ***	−0.238 (−0.426, −0.063)	0.004 **	−0.301 (−0.480, −0.126)	<0.001 ***	20.9 (7.5, 54.3)
② Trunk FM% (%)	−0.183 (−0.341, −0.048)	0.006 **	−0.820 (−1.570, −0.126)	0.022 *	−1.003 (−1.729, −0.296)	0.002 **	18.0 (4.4, 62.1)
(2) Trunk fat-free mass indices							
① Trunk FFM (kg)	−0.013 (−0.050, 0.021)	0.444	−0.124 (−0.252, −0.002)	0.046 *	−0.137 (−0.262, −0.019)	0.022 *	8.6 (−22.6, 76.1)
② Trunk FFM% (%)	0.188 (0.051, 0.340)	0.012 *	0.811 (0.062, 1.505)	0.028 *	0.998 (0.256, 1.708)	0.004 **	18.6 (4.7, 75.4)
(3) Trunk muscle mass indices							
① Trunk MM (kg)	−0.011 (−0.045, 0.020)	0.478	−0.124 (−0.246, −0.008)	0.042 *	−0.135 (−0.256, −0.022)	0.014 *	7.6 (−22.1, 65.1)
② Trunk MM% (%)	0.002 (0.001, 0.003)	0.008 **	0.007 (0.000, 0.014)	0.048 *	0.009 (0.002, 0.016)	0.004 **	20.1 (5.4, 87.8)
PCA-derived factors							
PCA_1	−0.152 (−0.282, −0.044)	0.006 **	−0.790 (−1.368, −0.252)	0.002 **	−0.942 (−1.500, −0.400)	<0.001 ***	16.1 (4.4, 42.6)

Abbreviations: BMI, body mass index; FM, fat mass; FM%, fat mass percentage; FMI, fat mass index; FFM, fat-free mass; FFM%, fat-free mass percentage; FFMI, fat-free mass index; MM, muscle mass; MM%, muscle mass percentage; PCA, principal component analysis. ^1^ Linear mixed effects models with school as a random intercept and adjusted for age, sex, and region. ^2^ Percentage of total effect mediated = [indirect effect/(indirect + direct effect)] × 100; 95% CI via 5000 bootstrap samples. *** *p* < 0.001, ** *p* < 0.01, * *p* < 0.05.

**Table 4:** Mediating role of screen time behaviour change in intervention effects on body composition.

Outcome	Indirect Effect ^1^ β (95% CI)	*p*	Direct Effect ^1^ β (95% CI)	*p*	Total Effect ^1^ β (95% CI)	*p*	% Mediated ^2^ (95% CI)
Whole-body composition							
(1) BMI (kg/m^2^)	−0.005 (−0.023, 0.008)	0.428	−0.394 (−0.612, −0.192)	<0.001 ***	−0.399 (−0.616, −0.194)	<0.001 ***	1.2 (−2.4, 6.9)
(2) Body fat mass indices							
① Body FM (kg)	−0.010 (−0.041, 0.015)	0.428	−0.640 (−1.000, −0.305)	<0.001 ***	−0.650 (−1.008, −0.311)	<0.001 ***	1.3 (−2.4, 7.9)
② Body FM% (%)	0.008 (−0.037, 0.056)	0.688	−1.011 (−1.663, −0.406)	<0.001 ***	−1.003 (−1.654, −0.389)	<0.001 ***	−0.8 (−7.5, 4.6)
③ Body FMI (kg/m^2^)	0.002 (−0.010, 0.016)	0.686	−0.297 (−0.454, −0.149)	<0.001 ***	−0.295 (−0.451, −0.146)	<0.001 ***	−0.7 (−6.4, 4.2)
(3) Body fat-free mass indices							
① Body FFM (kg)	−0.024 (−0.055, −0.002)	0.030 *	−0.174 (−0.391, 0.034)	0.114	−0.198 (−0.413, 0.010)	0.066	10.9 (−31.1, 96.5)
② Body FFM% (%)	−0.007 (−0.056, 0.037)	0.790	1.006 (0.356, 1.609)	<0.001 ***	0.999 (0.351, 1.612)	<0.001 ***	−0.5 (−6.8, 4.6)
③ Body FFMI (kg/m^2^)	−0.008 (−0.020, −0.001)	0.030 *	−0.091 (−0.200, 0.010)	0.094	−0.099 (−0.206, 0.003)	0.060	7.8 (−17.8, 62.5)
(4) Body muscle mass indices							
① Body MM (kg)	−0.023 (−0.053, −0.002)	0.028 *	−0.162 (−0.365, 0.033)	0.120	−0.185 (−0.386, 0.010)	0.066	11.1 (−32.1, 100.4)
② Body MM% (%)	0.000 (−0.001, 0.000)	0.734	0.010 (0.003, 0.015)	<0.001 ***	0.010 (0.003, 0.015)	<0.001 ***	−0.7 (−6.9, 4.3)
Trunk body composition							
(1) Trunk fat mass indices							
① Trunk FM (kg)	−0.008 (−0.026, 0.007)	0.268	−0.293 (−0.486, −0.113)	<0.001 ***	−0.301 (−0.493, −0.119)	<0.001 ***	2.5 (−2.2, 11.6)
② Trunk FM% (%)	0.003 (−0.052, 0.059)	0.886	−1.006 (−1.769, −0.299)	0.004 **	−1.003 (−1.767, −0.285)	0.004 **	−0.3 (−8.5, 7.3)
(2) Trunk fat-free mass indices							
① Trunk FFM (kg)	−0.013 (−0.032, 0.001)	0.068	−0.125 (−0.247, −0.006)	0.042 *	−0.138 (−0.259, −0.019)	0.018 *	9.0 (−1.7, 51.5)
② Trunk FFM% (%)	−0.001 (−0.057, 0.054)	0.998	0.997 (0.234, 1.704)	0.002 **	0.996 (0.233, 1.715)	0.002 **	0.0 (−7.6, 7.4)
(3) Trunk muscle mass indices							
① Trunk MM (kg)	−0.012 (−0.030, 0.000)	0.062	−0.124 (−0.241, −0.011)	0.032 *	−0.136 (−0.252, −0.023)	0.012 *	8.7 (−0.8, 47.0)
② Trunk MM% (%)	0.000 (−0.001, 0.001)	0.996	0.009 (0.002, 0.016)	0.002 **	0.009 (0.002, 0.016)	0.004 **	−0.0 (−8.2, 8.5)
PCA-derived factors							
PCA_1	0.005 (−0.040, 0.050)	0.812	−0.946 (−1.539, −0.398)	<0.001 ***	−0.942 (−1.533, −0.384)	<0.001 ***	−0.4 (−6.5, 5.2)

Abbreviations: BMI, body mass index; FM, fat mass; FM%, fat mass percentage; FMI, fat mass index; FFM, fat-free mass; FFM%, fat-free mass percentage; FFMI, fat-free mass index; MM, muscle mass; MM%, muscle mass percentage; PCA, principal component analysis. ^1^ Linear mixed effects models with school as a random intercept and adjusted for age, sex, and region. ^2^ Percentage of total effect mediated = [indirect effect/(indirect + direct effect)] × 100; 95% CI via 5000 bootstrap samples. *** *p* < 0.001, ** *p* < 0.01, * *p* < 0.05.

## Data Availability

The data presented in this study are available on request from the corresponding author due to ethical restrictions imposed by the Peking University Institutional Review Board. Requests to access the datasets should be directed to whjun@pku.edu.cn.

## Supplementary Materials

The following supporting information can be downloaded at: https://www.mdpi.com/article/10.3390/nu18183051/s1. Supplementary Materials File S1: Accelerometer subsample analysis; Table S1: Intervention effects on body composition and mediators in multivariate analysis adjusted for MVPA; Table S2: Mediating role of dietary behaviour change in intervention effects on body composition adjusted for MVPA.

## Abbreviations

The following abbreviations are used in this manuscript:

BIA	Bioelectrical impedance analyser
BMI	Body mass index
FFM	Fat-free mass
FFM%	Fat-free mass percentage
FFMI	Fat-free mass index
FM	Fat mass
FM%	Fat mass percentage
FMI	Fat mass index
MM	Muscle mass
MVPA	Moderate-to-vigorous physical activity
PCA	Principal component analysis

## References

[1] UNICEF https://news.un.org/zh/story/2025/09/1140658.

[2] Zhang X., Liu J., Ni Y., Yi C., Fang Y., Ning Q., Shen B., Zhang K., Liu Y., Yang L. (2024). Global Prevalence of Overweight and Obesity in Children and Adolescents: A Systematic Review and Meta-Analysis. JAMA Pediatr..

[3] Gao L., Peng W., Xue H., Wu Y., Zhou H., Jia P., Wang Y. (2023). Spatial-temporal trends in global childhood overweight and obesity from 1975 to 2030: A weight mean center and projection analysis of 191 countries. Glob. Health.

[4] World Obesity Atlas 2024. https://data.worldobesity.org/publications/WOF-Obesity-Atlas-v7.pdf.

[5] Lister N.B., Baur L.A., Felix J.F., Hill A.J., Marcus C., Reinehr T., Summerbell C., Wabitsch M. (2023). Child and adolescent obesity. Nat. Rev. Dis. Primers.

[6] Bjerregaard L.G., Jensen B.W., Ängquist L., Osler M., Sørensen T.I.A., Baker J.L. (2018). Change in Overweight from Childhood to Early Adulthood and Risk of Type 2 Diabetes. N. Engl. J. Med..

[7] Jebeile H., Kelly A.S., O’Malley G., Baur L.A. (2022). Obesity in children and adolescents: Epidemiology, causes, assessment, and management. Lancet Diabetes Endocrinol..

[8] Marcus C., Danielsson P., Hagman E. (2022). Pediatric obesity-Long-term consequences and effect of weight loss. J. Intern. Med..

[9] Ells L.J., Rees K., Brown T., Mead E., Al-Khudairy L., Azevedo L., McGeechan G.J., Baur L., Loveman E., Clements H. (2018). Interventions for treating children and adolescents with overweight and obesity: An overview of Cochrane reviews. Int. J. Obes..

[10] te Velde S.J., van Nassau F., Uijtdewilligen L., van Stralen M.M., Cardon G., De Craemer M., Manios Y., Brug J., Chinapaw M.J., ToyBox-Study Group (2012). Energy balance-related behaviours associated with overweight and obesity in preschool children: A systematic review of prospective studies. Obes. Rev..

[11] Coral D.E., Smit F., Farzaneh A., Gieswinkel A., Tajes J.F., Sparsø T., Delfin C., Bauvin P., Wang K., Temprosa M. (2025). Subclassification of obesity for precision prediction of cardiometabolic diseases. Nat. Med..

[12] Shao Y., Wang N., Shao M., Liu B., Wang Y., Yang Y., Li L., Zhong H. (2025). The lean body mass to visceral fat mass ratio is negatively associated with cardiometabolic disorders: A cross-sectional study. Sci. Rep..

[13] Manole L.M., Ghiga G., Iftinchi O., Boca L.O., Donos M.A., Tarca E., Ionut N., Revenco N., Margasoiu I., Trandafir L.M. (2025). Bioelectrical Impedance Analysis Versus Dual X-Ray Absorptiometry for Obesity Assessment in Pediatric Populations: A Systematic Review. Diagnostics.

[14] Luo R.H., Shen L.P., Zhang Q., Cao W., Wang H.L., Xu P.P., Yang Z.Y., Gan Q., Pang X.H., Xu T. (2024). Consistency between Bioelectrical Impedance Analysis and Dual-Energy X-Ray Absorptiometry in Body Composition Measurement in Children Aged 6–17 Years in China. Chin. J. Epidemiol..

[15] Pascu B.M., Bălănescu A., Bălănescu P.C., Delia C., Câmpean M., Gherghina I. (2025). Assessment of Insulin Resistance and Body Composition in Children with Overweight and Obesity: A Pilot Study Using Bioimpedance and Principal Component Analysis. Medicina.

[16] Liu Z., Wu Y., Niu W.Y., Feng X., Lin Y., Gao A., Zhang F., Fang H., Gao P., Li H.J. (2019). A school-based, multi-faceted health promotion programme to prevent obesity among children: Protocol of a cluster-randomised controlled trial (the DECIDE-Children study). BMJ Open.

[17] Liu Z., Gao P., Gao A.Y., Lin Y., Feng X.X., Zhang F., Xu L.Q., Niu W.Y., Fang H., Zhou S. (2022). Effectiveness of a Multifaceted Intervention for Prevention of Obesity in Primary School Children in China A Cluster Randomized Clinical Trial. JAMA Pediatr..

[18] Wang H.X., Cheng L., Yuan X., Lyu J.L., Li P., Yan S.Y., Wang H., Ding Y.S., Hong S.D., Wang H.J. (2025). The Mediating Effect of Concurrent Changes in Dietary Behaviors on the Associations Between Intervention and Changes in Adiposity Outcomes: Evidence from a Cluster-Randomized Controlled Trial. Nutrients.

[19] Verney J., Metz L., Chaplais E., Cardenoux C., Pereira B., Thivel D. (2016). Bioelectrical Impedance Is an Accurate Method to Assess Body Composition in Obese but Not Severely Obese Adolescents. Nutr. Res..

[20] Thivel D., Verney J., Miguet M., Masurier J., Cardenoux C., Lambert C., Courteix D., Metz L., Pereira B. (2018). The Accuracy of Bioelectrical Impedance to Track Body Composition Changes Depends on the Degree of Obesity in Adolescents with Obesity. Nutr. Res..

[21] Uçar M.K., Uçar K., Uçar Z., Bozkurt M.R. (2022). Determination gender-based hybrid artificial intelligence of body muscle percentage by photoplethysmography signal. Comput. Methods Programs Biomed..

[22] Ramírez Torres M., Ruiz Valenzuela R.E., Esparza-Romero J., López Teros M.T., Alemán-Mateo H. (2019). The fat mass index, not the fat-free mass index, is associated with impaired physical performance in older adult subjects: Evidence from a cross-sectional study. Clin. Nutr..

[23] Wang R., Xu Y., Zhang Y., Wu Y., Zuo A., Liu S., Ba Y., Xu H., Weng S., Zhou Z. (2025). Total and regional fat-to-muscle mass ratio and risks of pan-cancer: A prospective cohort study. BMC Med..

[24] Lee P.H., Macfarlane D.J., Lam T.H., Stewart S.M. (2011). Validity of the International Physical Activity Questionnaire Short Form (IPAQ-SF): A systematic review. Int. J. Behav. Nutr. Phys. Act..

[25] World Health Organization Global Recommendations on Physical Activity for Health 2010. https://www.who.int/publications/i/item/9789241599979.

[26] Su X., Hassan M.A., Kim H., Gao Z. (2025). Comparative effectiveness of lifestyle interventions on children’s body composition management: A systematic review and network meta-analysis. J. Sport. Health Sci..

[27] Kamil N.Z.I.A., Mokhtar A.H., Yahya A., Zain F.M., Selamat R., Ishak Z., Jalaludin M.Y. (2025). Effects of the MyBFF@school program on anthropometry and body composition among overweight and obese adolescent schoolchildren. BMC Public Health.

[28] Minja E.G., Mrimi E.C., Mponzi W.P., Beckmann J., Finda M.F., Okumu F.O., Lang C., Gerber M., Utzinger J., Long K.Z. (2025). Effect of physical activity and multi-micronutrient supplementation on body composition among Tanzanian schoolchildren: Secondary outcomes from the KaziAfya cluster-randomized controlled trial. BMC Public Health.

[29] Hu C., Zhang J., Lin L., Jia X., Wang S., Zheng R., Li M., Xu Y., Xu M., Lin H. (2026). Associations of general and central obesity with risk of premature mortality among Chinese adults: A nationwide prospective cohort study. Diabetes Obes. Metab..

[30] Re F., Oguntade A.S., Bohrmann B., Bragg F., Carter J.L. (2023). Associations of general and central adiposity with hypertension and cardiovascular disease among South Asian populations: A systematic review and meta-analysis. BMJ Open.

[31] Kim M.Y., An S., Shim Y.S., Lee H.S., Hwang J.S. (2024). Waist-height ratio and body mass index as indicators of obesity and cardiometabolic risk in Korean children and adolescents. Ann. Pediatr. Endocrinol. Metab..

[32] Aucouturier J., Meyer M., Thivel D., Taillardat M., Duché P. (2009). Effect of android to gynoid fat ratio on insulin resistance in obese youth. Arch. Pediatr. Adolesc. Med..

[33] Dao H.H., Frelut M.L., Oberlin F., Peres G., Bourgeois P., Navarro J. (2004). Effects of a multidisciplinary weight loss intervention on body composition in obese adolescents. Int. J. Obes. Relat. Metab. Disord..

[34] Cohen T.R., Mak I.L., Loiselle S.E., Kasvis P., Hazell T.J., Vanstone C.A., Rodd C., Weiler H.A. (2023). Changes in Adiposity without Impacting Bone Health in Nine- to Twelve-Year-Old Children with Overweight and Obesity after a One-Year Family-Centered Lifestyle Behavior Intervention. Child. Obes..

[35] Dixon H., Apperley L., Senniappan S., Parkinson J. (2024). The effect of lifestyle and pharmacological interventions on body composition in childhood obesity. Endocr. Abstr..

[36] Tangtongsoong A., Visuthranukul C., Chongpison Y., Chomtho S. (2025). Differential effects of dietary and physical activity interventions on adiposity of children with obesity. Clin. Exp. Pediatr..

[37] Najafi M., Hariri Z., Nikpayam O., Sarvi A.H., Pasand M., Noura P., Farajian Nejad F. (2025). The effects of low carbohydrate and high protein diet on the anthropometric indices, blood pressure, metabolic factors, and hormones related to metabolism: A systematic review and meta-analysis. Diabetes Metab. Syndr. Clin. Res. Rev..

[38] Liu Z., Yue Z.H., Wen L.M., Zhao J.F., Zhou S., Gao A.Y., Zhang F., Wang H.J. (2024). Time-specific intervention effects on objectively measured physical activity in school children. J. Public Health.

[39] Hsueh M.C., Chen Y.H. (2025). Associations of physical activity intensity and specific sedentary behaviors during after-school and weekend with overweight and obesity among preschool children. BMC Pediatr..

[40] Matke A., Olds T., Maher C., Fraysse F., Dumuid D. (2025). Cross-sectional and longitudinal associations between sleep, sedentary behaviour and physical activity with adiposity and cardio-respiratory fitness in school-aged children: A compositional data analysis. J. Act. Sedentary Sleep Behav..

[41] Alkhatib A., Obita G. (2024). Childhood Obesity and Its Comorbidities in High-Risk Minority Populations: Prevalence, Prevention and Lifestyle Intervention Guidelines. Nutrients.

[42] Obita G., Alkhatib A. (2023). Effectiveness of Lifestyle Nutrition and Physical Activity Interventions for Childhood Obesity and Associated Comorbidities among Children from Minority Ethnic Groups: A Systematic Review and Meta-Analysis. Nutrients.

[43] Neil-Sztramko S.E., Caldwell H., Dobbins M. (2021). School-based physical activity programs for promoting physical activity and fitness in children and adolescents aged 6 to 18. Cochrane Database Syst. Rev..

[44] Livingstone M.B., Robson P.J. (2000). Measurement of Dietary Intake in Children. Proc. Nutr. Soc..

[45] Slootmaker S.M., Schuit A.J., Chinapaw M.J., Seidell J.C., van Mechelen W. (2009). Disagreement in Physical Activity Assessed by Accelerometer and Self-Report in Subgroups of Age, Gender, Education and Weight Status. Int. J. Behav. Nutr. Phys. Act..

[46] Sriprom D., Buranarugsa R. (2025). A Study of Body Composition and Prevalence of Obesity in Grade 6 Demonstration School Students, Khon Kaen University. J. Intellect. Educ..

