# Supplementary material for: Dietary Behaviours Mediate Intervention Effects on Body Composition Outcomes in Children: Evidence from a Cluster-Randomised Controlled Trial
Source: Nutrients. 2026 Sep 18;18(18):3051. doi: 10.3390/nu18183051 (PMC13610272; doi:10.3390/nu18183051)
Supplement: Supplementary file 1 [file nutrients-18-03051-s001.zip › Supplementary Materials File S1. Accelerometer Subsample Analysis.pdf]

Accelerometer Subsample Analysis: In-School MVPA (n=88)

Accelerometer-measured MVPA as a continuous variable:

Using the same covariates as in the main analysis (adjusted for age, sex, and region), we examined the intervention effect on in-school MVPA (minutes/hour during weekdays, 8:00 AM – 4:59 PM) as a continuous variable in the accelerometer subsample. The results showed no significant between-group difference ($\beta = 0.4267$, 95%CI: −0.411 to 1.264, $p = 0.247$).

```
            Estimate Std. Error      df t value Pr(>|t|)
group         0.4267     0.3257  5.0011   1.310   0.2472
```
