# Supplementary material for: Dietary Behaviours Mediate Intervention Effects on Body Composition Outcomes in Children: Evidence from a Cluster-Randomised Controlled Trial
Source: Nutrients. 2026 Sep 18;18(18):3051. doi: 10.3390/nu18183051 (PMC13610272; doi:10.3390/nu18183051)
Supplement: Supplementary file 1 [file nutrients-18-03051-s001.zip › Table S1. Intervention Effects on Body Composition and Mediators in Multivariate Analysis Adjusted for MVPA.pdf]

Table S1. Intervention Effects on Body Composition and Mediators in Multivariate Analysis Adjusted for MVPA.

Outcome	Multivariate analysis [1] β (95 % CI) [2]	*p*
Whole body composition		
(1) BMI (kg/m^2)	−0.398 (−0.608, −0.188)	0.002 **
(2) Body fat mass indices		
① Body FM (kg)	−0.643 (−0.986, −0.299)	0.002 **
② Body FM % (%)	−1.000 (−1.622, −0.378)	0.006 **
③ Body FMI (kg/m^2)	−0.293 (−0.443, −0.143)	0.001 **
(3) Body fat-free mass indices		
① Body FFM (kg)	−0.196 (−0.405, 0.014)	0.090
② Body FFM % (%)	0.997 (0.377, 1.617)	0.006 **
③ Body FFMI (kg/m^2)	−0.100 (−0.204, 0.005)	0.083
(4) Body muscle mass indices		
① Body MM (kg)	−0.183 (−0.379, 0.013)	0.090
② Body MM % (%)	0.009 (0.004, 0.015)	0.005 **
Trunk body composition		
(1) Trunk fat mass indices		
① Trunk FM (kg)	−0.298 (−0.482, −0.114)	0.006 **
② Trunk FM % (%)	−1.005 (−1.738, −0.271)	0.016 *
(2) Trunk fat-free mass indices		
① Trunk FFM (kg)	−0.135 (−0.254, −0.017)	0.041 *
② Trunk FFM % (%)	0.998 (0.265, 1.731)	0.017 *
(3) Trunk muscle mass indices		
① Trunk MM (kg)	−0.135 (−0.248, −0.022)	0.033 *
② Trunk MM % (%)	0.009 (0.002, 0.016)	0.022 *
PCA-derived factors		
PCA_1	−0.940 (−1.514, −0.366)	0.005 **
Candidate mediators		
Dietary behaviour change (0–5)	1.074 (0.763, 1.385)	<0.001 ***
Screen-time target attainment [3]	1.852 (1.298, 2.643)	0.001 **

Abbreviations: BMI, body mass index; FM, fat mass; FM%, fat mass percentage; FMI, fat mass index; FFM, fat free mass; FFM%, fat free mass percentage; FFMI, fat free mass index; MM, muscle mass; MM%, muscle mass percentage; MVPA, moderate-to-vigorous physical activity; PCA, principal component analysis.

[1] Linear mixed-effects models with school as a random intercept and adjusted for age, sex, region and baseline MVPA.

[2] Values are β coefficients (95 % CI).

[3] Dichotomised as 1 = meets guideline, 0 = does not meet guideline.

*** $p < 0.001$, ** $p < 0.01$, * $p < 0.05$.
