# Supplementary material for: Dietary Behaviours Mediate Intervention Effects on Body Composition Outcomes in Children: Evidence from a Cluster-Randomised Controlled Trial
Source: Nutrients. 2026 Sep 18;18(18):3051. doi: 10.3390/nu18183051 (PMC13610272; doi:10.3390/nu18183051)
Supplement: Supplementary file 1 [file nutrients-18-03051-s001.zip › Table S2. Mediating Role of Dietary Behaviour Change in Intervention Effects on Body Composition Adjusted for MVPA.pdf]

Table S2. Mediating Role of Dietary Behaviour Change in Intervention Effects on Body Composition Adjusted for MVPA.

Outcome	Indirect effect [1] β (95 % CI)	*p*	Direct effect [1] β (95 % CI)	*p*	Total effect [1] β (95 % CI)	*p*	% mediated [2] (95 % CI)
Whole body composition							
(1) BMI (kg/m²)	–0.054 (–0.094, –0.017)	0.002 **	–0.344 (–0.559, –0.130)	0.002 **	–0.398 (–0.610, –0.187)	<0.001 ***	13.2 (4.1, 33.2)
(2) Body fat mass indices							
① Body FM (kg)	–0.110 (–0.183, –0.041)	<0.001 ***	–0.534 (–0.879, –0.187)	0.002 **	–0.643 (–0.983, –0.301)	<0.001 ***	16.5 (6.1, 40.0)
② Body FM % (%)	–0.155 (–0.277, –0.046)	0.006 **	–0.846 (–1.483, –0.210)	0.014 *	–1.001 (–1.627, –0.375)	0.002 **	14.9 (3.8, 45.7)
③ Body FMI (kg/m²)	–0.047 (–0.082, –0.016)	<0.001 ***	–0.246 (–0.397, –0.094)	0.002 **	–0.294 (–0.441, –0.143)	<0.001 ***	15.7 (5.2, 37.9)
(3) Body fat-free mass indices							
① Body FFM (kg)	–0.034 (–0.087, 0.018)	0.194	–0.161 (–0.386, 0.065)	0.148	–0.196 (–0.413, 0.027)	0.080	15.0 (–87.5, 115.4)
② Body FFM % (%)	0.151 (0.042, 0.283)	0.002 **	0.848 (0.212, 1.482)	0.006 **	0.999 (0.396, 1.607)	<0.001 ***	15.1 (3.9, 44.1)
③ Body FFMI (kg/m²)	–0.018 (–0.037, 0.000)	0.058	–0.082 (–0.194, 0.030)	0.132	–0.100 (–0.210, 0.010)	0.078	15.7 (–73.3, 101.9)
(4) Body muscle mass indices							
① Body MM (kg)	–0.031 (–0.081, 0.017)	0.200	–0.152 (–0.361, 0.060)	0.142	–0.183 (–0.386, 0.025)	0.080	14.7 (–86.8, 117.9)
② Body MM % (%)	0.001 (0.000, 0.003)	0.002 **	0.008 (0.002, 0.014)	0.006 **	0.010 (0.004, 0.015)	<0.001 ***	15.4 (4.2, 43.9)
Trunk body composition							
(1) Trunk fat mass indices							
① Trunk FM (kg)	–0.063 (–0.105, –0.025)	<0.001 ***	–0.235 (–0.419, –0.050)	0.016 *	–0.298 (–0.478, –0.115)	0.002 **	20.5 (7.3, 58.4)
② Trunk FM % (%)	–0.183 (–0.325, –0.055)	0.004 **	–0.822 (–1.575, –0.071)	0.032 *	–1.006 (–1.746, –0.265)	0.010 *	17.3 (4.2, 67.0)
(2) Trunk fat-free mass indices							
① Trunk FFM (kg)	–0.013 (–0.048, 0.023)	0.468	–0.122 (–0.248, 0.006)	0.062	–0.135 (–0.254, –0.010)	0.030 *	9.1 (–28.5, 75.4)

② Trunk FFM % (%)	0.186 (0.058, 0.339)	0.002 **	0.814 (0.062, 1.565)	0.030 *	1.000 (0.286, 1.719)	0.004 **	18.5 (4.9, 67.0)
(3) Trunk muscle mass indices							
① Trunk MM (kg)	–0.011 (–0.044, 0.021)	0.498	–0.124 (–0.243, –0.002)	0.050	–0.135 (–0.249, –0.015)	0.026 *	8.0 (–24.8, 63.8)
② Trunk MM % (%)	0.002 (0.001, 0.003)	0.002 **	0.007 (0.000, 0.014)	0.046 *	0.009 (0.002, 0.016)	0.006 **	20.3 (5.4, 82.7)
PCA-derived factors							
PCA_1	–0.142 (–0.257, –0.036)	0.012 *	–0.799 (–1.382, –0.210)	0.014 *	–0.941 (–1.515, –0.365)	0.002 **	14.6 (3.4, 44.0)

Abbreviations: BMI, body mass index; FM, fat mass; FM%, fat mass percentage; FMI, fat mass index; FFM, fat free mass; FFM%, fat free mass percentage; FFMI, fat free mass index; MM, muscle mass; MM%, muscle mass percentage; MVPA, moderate-to-vigorous physical activity; PCA, principal component analysis.

[1] Linear mixed-effects models with school as a random intercept and adjusted for age, sex, region and baseline MVPA.

[2] Percentage of total effect mediated = [indirect effect / (indirect + direct effect)] × 100; 95 % CI via 5,000 bootstrap samples.

*** $p < 0.001$, ** $p < 0.01$, * $p < 0.05$.
